# Transcriptional responses to glucose in *Saccharomyces cerevisiae *strains lacking a functional protein kinase A

**DOI:** 10.1186/1471-2164-12-405

**Published:** 2011-08-09

**Authors:** Daniela Livas, Marinka JH Almering, Jean-Marc Daran, Jack T Pronk, Juana M Gancedo

**Affiliations:** 1 Department of Metabolism and Cell Signalling, Instituto de Investigaciones Biomédicas Alberto Sols, CSIC-UAM, Arturo Duperier 4, 28029 Madrid, Spain; 2 Department of Biotechnology, Delft University of Technology and Kluyver Centre for Genomics and Industrial Fermentation, Julianalaan 67, 2628 BC Delft, The Netherlands

## Abstract

**Background:**

The pattern of gene transcripts in the yeast *Saccharomyces cerevisiae *is strongly affected by the presence of glucose. An increased activity of protein kinase A (PKA), triggered by a rise in the intracellular concentration of cAMP, can account for many of the effects of glucose on transcription. In *S. cerevisiae *three genes, *TPK1, TPK2*, and *TPK3*, encode catalytic subunits of PKA. The lack of viability of *tpk1 tpk2 tpk3 *triple mutants may be suppressed by mutations such as *yak1 *or *msn2/msn4*. To investigate the requirement for PKA in glucose control of gene expression, we have compared the effects of glucose on global transcription in a wild-type strain and in two strains devoid of PKA activity, *tpk1 tpk2 tpk3 yak1 *and *tpk1 tpk2 tpk3 msn2 msn4*.

**Results:**

We have identified different classes of genes that can be induced -or repressed- by glucose in the absence of PKA. Representative examples are genes required for glucose utilization and genes involved in the metabolism of other carbon sources, respectively. Among the genes responding to glucose in strains devoid of PKA some are also controlled by a redundant signalling pathway involving PKA activation, while others are not affected when PKA is activated through an increase in cAMP concentration. On the other hand, among genes that do not respond to glucose in the absence of PKA, some give a full response to increased cAMP levels, even in the absence of glucose, while others appear to require the cooperation of different signalling pathways. We show also that, for a number of genes controlled by glucose through a PKA-dependent pathway, the changes in mRNA levels are transient. We found that, in cells grown in gluconeogenic conditions, expression of a small number of genes, mainly connected with the response to stress, is reduced in the strains lacking PKA.

**Conclusions:**

In *S. cerevisiae*, the transcriptional responses to glucose are triggered by a variety of pathways, alone or in combination, in which PKA is often involved. Redundant signalling pathways confer a greater robustness to the response to glucose, while cooperative pathways provide a greater flexibility.

## Background

Adaptation to changing environmental conditions is of capital importance for the survival of organisms and their successful establishment in new ecological niches. To enable adaptation, sophisticated networks of signalling that govern growth and development have been selected in all living systems. In the model yeast *Saccharomyces cerevisiae*, availability of glucose or other easily fermentable sugars elicits a drastic rearrangement of metabolism and multiple changes in its transcriptome. An immediate consequence of the exposure to glucose is a rise in the concentration of internal cAMP [1,2], and it is well established that changes in cAMP levels affect the transcription rate of a large number of genes in *S. cerevisiae *[3-5]. The increase in cAMP levels is mainly due to the activation of the plasma membrane-bound adenylate cyclase mediated by different G proteins, Ras1, Ras2 and Gpa2 [6,7]. The GDP-GTP exchange in the Ras1 or Ras2 proteins is mediated by Cdc25 [8], which is activated by glucose by an, as yet, unknown mechanism [9]. Activation of Gpa2, in turn, results from the binding of glucose to the transmembrane protein Gpr1 [10-12]. Bcy1, the regulatory subunit of the cAMP-dependent protein kinase (PKA), when binding cAMP, dissociates from the catalytic subunits and thereby activates them. In a yeast strain harbouring a *RAS2^Val19 ^*gene, encoding a constitutively active form of Ras2, under the control of the *GAL1 *promoter, an increase in internal cAMP levels may be achieved in the absence of glucose through the addition of galactose. Even when galactose metabolism in such a strain is blocked by disruption of the *GAL1 *gene (encoding galactokinase), the increase in cAMP levels that follows galactose addition causes many of the effects on transcription that are elicited by glucose [5]. The same authors also report that in a *bcy1 *strain, where PKA activity is independent of cAMP levels, transcription of many genes still responds to the presence of glucose. This last observation suggests that there are additional, and possibly redundant, glucose signalling pathway(s) independent of changes in PKA activity triggered by cAMP [5]. This does not, however, discard the possibility that the alternative pathway(s) require a basal activity of PKA, even if they do not depend on its activation by cAMP.

**Figure 1:**
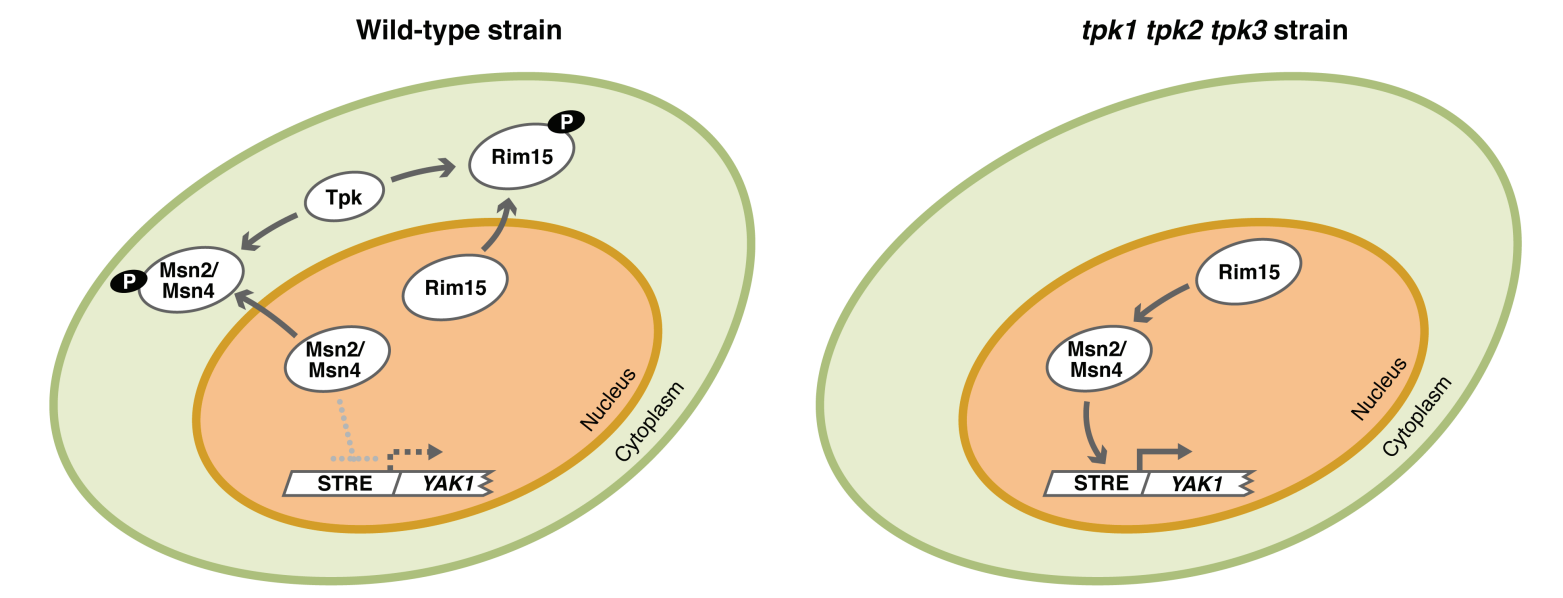
**Relationships between Tpk1, 2, 3, Rim15, Msn2/4 and Yak1**. Activated Tpks can phosphorylate Msn2/4 and Rim15 leading to their export to the cytoplasm, thus reducing the transcription rate of *YAK1 *and allowing growth. In the absence of Tpks, Rim15 and Msn2/4 are not phosphorylated and remain in the nucleus, in an active or potentially active form. Rim15 activates Msn2/4, Msn2/4 activate the transcription of *YAK1 *and Yak1 blocks cellular growth.

The goal of the present study was to investigate the occurrence of PKA-independent glucose signalling in *S. cerevisiae*. To this end, we have used global transcription analysis to study the effects of glucose on yeast strains completely devoid of PKA activity. In *S. cerevisiae *three genes *TPK1, TPK2*, and *TPK3 *encode catalytic subunits of PKA. While strains expressing only one of these genes grow normally, a triple null mutant (*tpk1 tpk2 tpk3*) is not viable [13]. Characterization of different mutations able to suppress the growth defect of the triple mutant [14-16] has enabled identification of the crucial function of PKA. As shown in Figure 1, PKA is needed to counteract the negative effect of the protein kinase Yak1 on yeast growth [17,18]. In the presence of PKA the protein kinase Rim15 [15] and the transcription factors Msn2 and Msn4 [19] can be phosphorylated and exported to the cytoplasm. As a consequence, transcription of Msn2/Msn4 activated genes, among them *YAK1 *[16], is reduced, Yak1 levels remain low and growth is not hindered. In the absence of PKA, Rim15 remains in the nucleus where it can activate Msn2/Msn4 [20] that turn on *YAK1 *transcription. Yak1 then blocks growth by mechanisms that may include the phosphorylation and activation of Msn2/Msn4 [21]. This explains why strains lacking Rim15, Msn2/Msn4 or Yak1 no longer require PKA for growth. In this work we have used two isogenic strains lacking PKA and carrying the suppressor mutations *msn2 msn4 *or *yak1*. Two different suppressor mutants were used with the aim to enable a dissection of effects of the lack of PKA and effects of the suppressor mutations themselves.

We found that glucose induction of genes related with glucose metabolism or glucose repression of genes involved in the utilization of carbon sources alternative to glucose can proceed in the absence of PKA. We noted, however, that genes related with similar types of processes, such as biosynthesis of amino acids or transport of drugs, may respond differently to the lack of PKA.

## Results

### Genes induced by glucose

**Figure 2:**
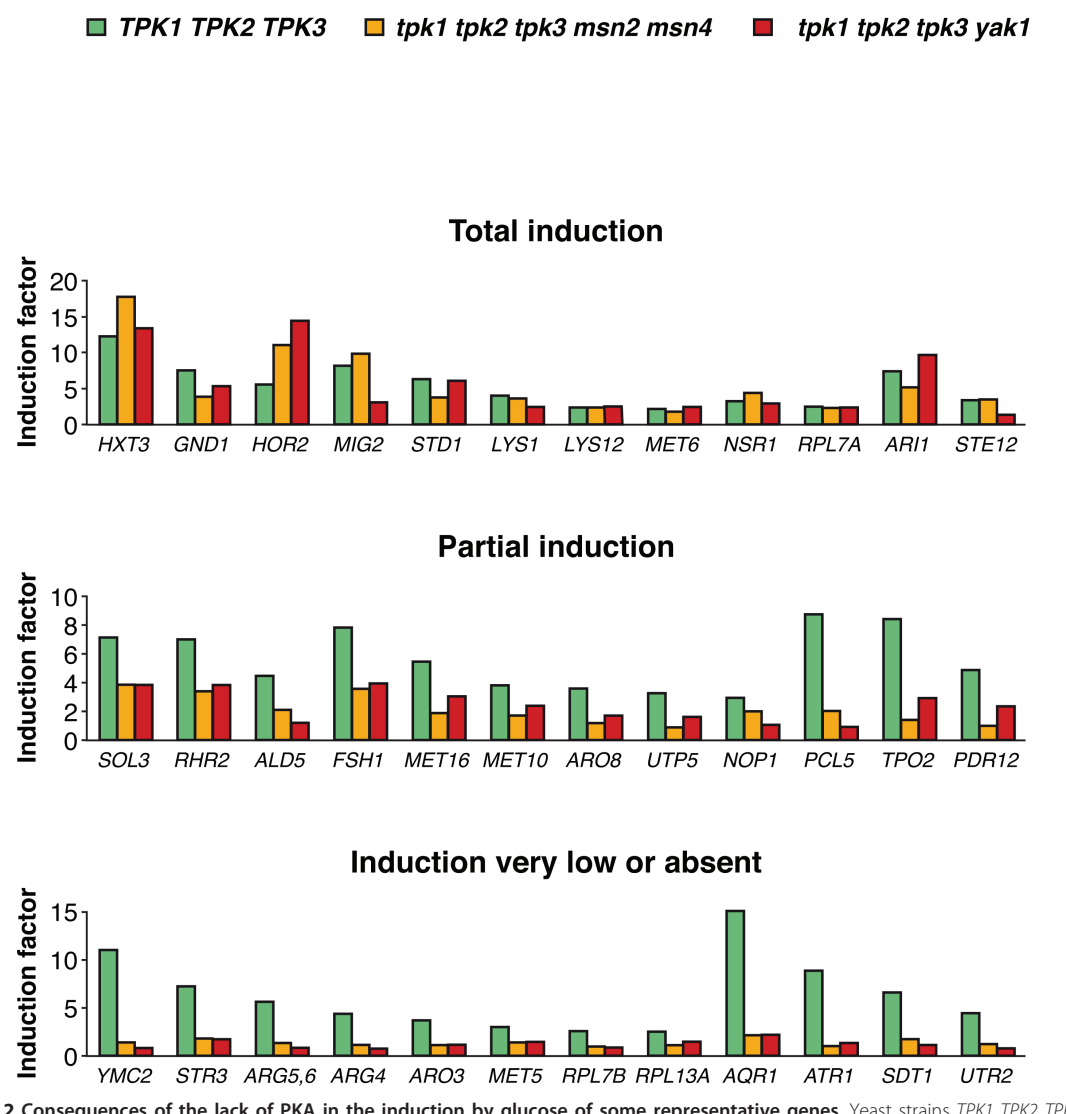
**Consequences of the lack of PKA in the induction by glucose of some representative genes**. Yeast strains *TPK1 TPK2 TPK3, tpk1 tpk2 tpk3 msn2 msn4*, and *tpk1 tpk2 tpk3 yak1 *were grown on ethanol and samples were taken before the addition of glucose to the medium and 30 min after, as described in Methods. mRNA levels were measured using Affymetrix microarrays. Biological duplicates were performed. Expression data from biological replicates generally differed from the average by less than 25%. The induction factor is the quotient of the values measured in samples incubated in the presence of glucose and in those grown in the absence of glucose. Induction is considered "total" when the induction factor is no less than 70% of that measured in the reference strain in at least one of the strains lacking PKA.

When ethanol-grown cultures of the *TPK1 TPK2 TPK3 *reference strain were exposed to glucose over 700 genes showed at least a 1.8-fold increase of their transcript level and ca. 80 genes were induced by more than 4-fold. Among the genes induced by glucose, some were still strongly induced in at least one of the strains without functional *TPK *genes (induction factor of at least 70% of that measured in the reference strain), others were only partially induced, and still others did not show a transcriptional response to glucose (representative genes of each category are shown in Figure 2).

For some genes, such as *MIG2*, the induction factor was different in the two strains lacking PKA (Figure 2). However, the observation that full induction takes place in one of the two strains was sufficient to indicate the existence of a glucose-signalling pathway independent of PKA. For genes such as *HOR2*, PKA may even attenuate the degree of glucose induction, which for this gene was higher in both strains lacking PKA (Figure 2).

Genes encoding proteins related with glucose metabolism (*HXT3, PFK1, ENO1, ENO2, CDC19, PDC1, ADH1, GND1, TKL1, HOR2, PMA1*) mainly showed PKA-independent glucose induction, although in some cases the induction ratios decreased in the absence of PKA (*ALD5, HXT1, PDC5, SOL3, RHR2*) (Figure 2 and results not shown). Genes encoding diverse proteins that regulate carbon metabolism were also well induced in the absence of PKA (*STD1, MIG2, MIG3*) or partially induced (*GCR1*). For other categories of genes the results were not as clear-cut. For instance, for genes encoding proteins involved in amino acid biosynthesis we found that those in the lysine pathway (*LYS1, LYS9, LYS12, LYS20*) mostly showed PKA-independent glucose induction (with *LYS21 *as a notable exception), while the genes related with the arginine pathway (*ARG1, ARG3, ARG4, ARG5,6, ARG7*) were not induced by glucose in strains lacking PKA. Among the methionine biosynthesis genes, *MET3, MET6 *or *MET32 *did not require PKA for glucose induction, while in the absence of PKA induction was reduced for *MET10, MET13 *or *MET16 *and absent for *MET5*. Similarly, while most genes encoding ribosomal proteins and proteins involved in ribosome biogenesis (*RPL4B, RPL14B, RPL16A, DBD2, DBP8, NOG1, NOP16, NSR1 *and many more) still responded to glucose in the absence of PKA, expression of four of them (*RPL7B, RPL13A, RPS22B, NHP2*) was not induced by glucose in the PKA-negative strains. Among multidrug membrane transporters, *ATR1 *was not induced by glucose in the absence of PKA, *AQR1 *was much less induced than in the wild-type strain and for *PDR12 *a 2-3-fold decrease in the induction level was observed.

### Effects of PKA on induction by glucose during exponential growth

For some yeast genes, induction by glucose is a transient phenomenon [5]. We, therefore, compared short-term induction by glucose and expression during exponential growth on glucose for a subset of genes, in strains with and without PKA (Table 1). To discard the possibility that differences could depend on repressing or inducing effects of ethanol, we tested cultures growing on glucose in the presence of ethanol. In the *TPK1,2,3 *reference strain we observed a strong induction of *PDC1 *by glucose, both in the short-term experiments and during exponential growth. For the other genes tested, induction ratios were more pronounced in the short-term experiments, an effect that was particularly marked for *PDC5 *(Table 1).

**Table 1:** Short-term vs. Long-term effects of lack of PKA on induction of different genes by glucose

Strain	Growth conditions	Gene					
		
		*PDC1*	*PDC5*	*ALD5*	*TMT1*	*YMC2*	*ATR1*
		
		Induction factor					
TPK1 TPK2 TPK3	30 min with glucose	12.8	235	5	9.7	11	13.8
	
	growth in medium with glucose	27	1.3	3	2	4	2.3

*tpk1 tpk2 tpk3* *msn2 msn4*	30 min with glucose	15	70	3.4	1.7	2.2	2.6
	
	growth in medium with glucose	21	4.3	3.2	1.9	1.9	2.9

*tpk1 tpk2 tpk3* *yak1*	30 min with glucose	1.2	1.8	0.9	1.1	0.8	1.4
	
	growth in medium with glucose	4.4	2	0.6	0.7	0.7	0.9

For short-term experiments yeasts were grown on ethanol as described in Methods and samples were taken before the addition of glucose to the medium and 30 min after. The yeasts were also grown on a medium containing 2% ethanol and 4% glucose and collected when over 50% of the glucose was still present in the medium. Biological duplicates were performed and mRNA levels were measured by RT-PCR as described. Expression data from biological replicates generally differed from the average by less than 25%. The induction factor is the quotient of the values measured in samples grown in the presence of glucose and in the absence of glucose.

In the *msn2 msn4 *background, the absence of PKA had no effect on the induction of *PDC1*, while for *PDC5 *and *ALD5 *only a weak effect in the short-term experiments was observed. For *TMT1, YMC2 *and *ATR1 *short-term induction was much reduced in the *tpk1 tpk2 tpk3 msn2 msn4 *strain, but a 2-3-fold induction was still seen during exponential growth on glucose, similar to that observed in the wild-type strain. In the *tpk1 tpk2 tpk3 yak1 *strain, glucose induction was very much reduced for all six genes and absent in most cases (Table 1). This suggests a cooperative effect of the absence of PKA and the absence of Yak1, as all six genes were normally induced by glucose, although sometimes with a delay, in a *yak1 *strain (results not shown).

### Genes repressed by glucose

**Figure 3:**
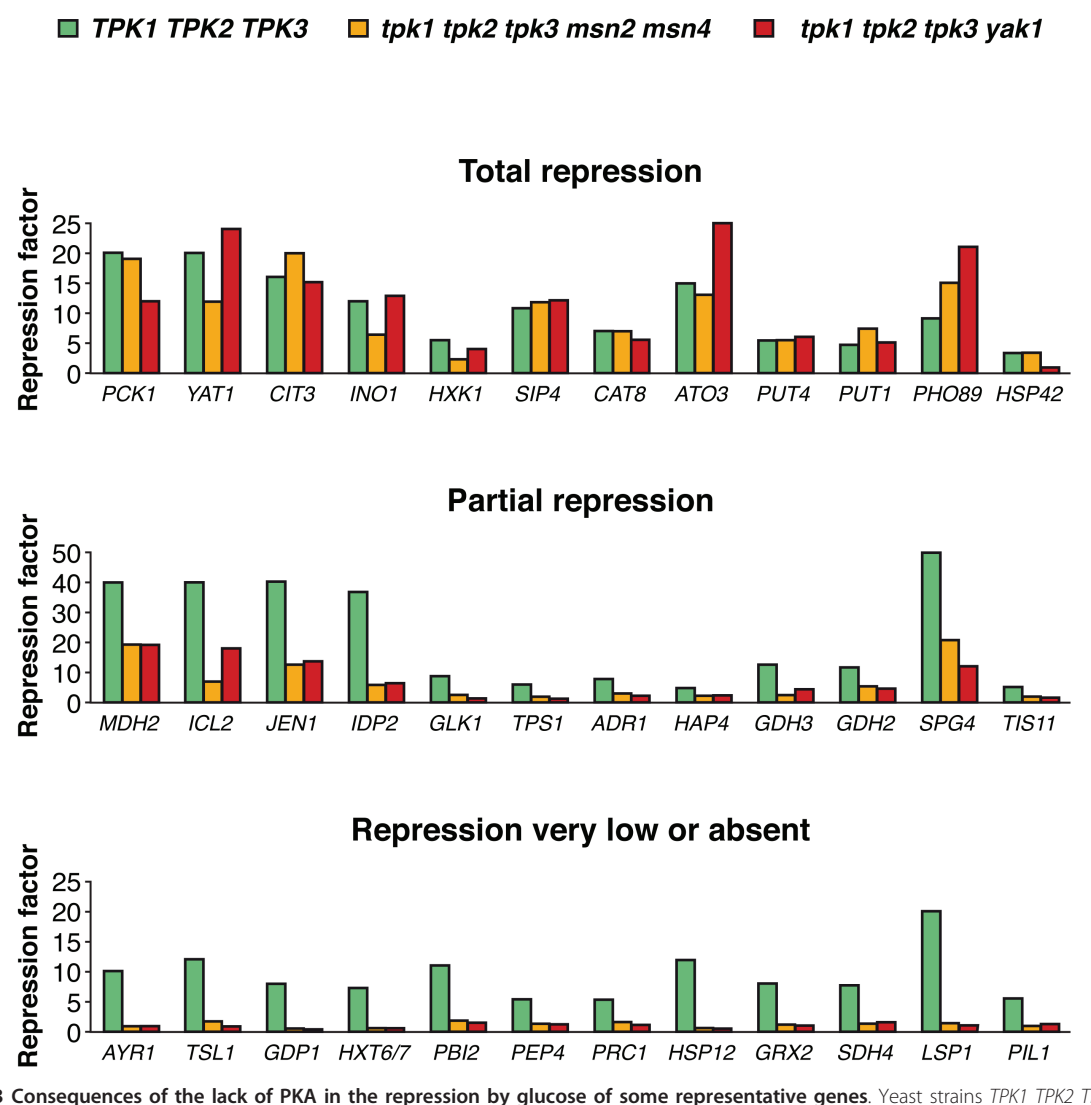
**Consequences of the lack of PKA in the repression by glucose of some representative genes**. Yeast strains *TPK1 TPK2 TPK3, tpk1 tpk2 tpk3 msn2 msn4*, and *tpk1 tpk2 tpk3 yak1 *were grown on ethanol and samples were taken before the addition of glucose to the medium and 30 min after, as described in Methods. mRNA levels were measured using Affymetrix microarrays. Biological duplicates were performed. Expression data from biological replicates generally differed from the average by less than 25%. The repression factor is the quotient of the values measured in samples grown in the absence of glucose and in those incubated in the presence of glucose. Repression is considered "total" when the repression factor is no less than 70% of that measured in the reference strain in at least one of the strains lacking PKA.

Exposure to glucose causes transcriptional repression of a large number of *S. cerevisiae *genes. In the present study, short-term exposure to glucose of the *TPK1 TPK2 TPK3 *reference strain caused the repression of 500 genes by at least 4-fold and of 70 genes by at least 15-fold. As we observed when studying glucose induction, some of these genes were also strongly repressed (repression factor of at least 70% of that measured in the reference strain) in strains without a functional PKA, others were partially repressed, and still others not repressed at all in the PKA-negative strains. Representative expression profiles for each of these groups are shown in Figure 3.

Many of the genes required for growth on non-fermentable carbon sources were repressed by glucose in at least one of the two yeast strains without a functional PKA, either fully (*ACS1, ADY2, CIT2, CIT3, DLD1, FBP1, ICL1, MDH2, MLS1, PCK1, SFC1*) or partially (*ACH1, ACO1, ADH2, CIT1, COX7, CYB2, CYT1, FUM1, GUT2, IDH1, IDP2, JEN1, KGD1, KGD2, LSC1, MDH1, MDH3*) (Figure 3 and results not shown). Besides, genes encoding proteins that control carbon metabolism were repressed by glucose in the absence of PKA, with some showing full (*CAT8, MAL33, REG2, SIP4, SNF3*) and others partial (*ADR1, HAP4, RGT1*) repression.

Several genes encoding proteins with a variety of other functions were also strongly repressed by glucose in the absence of PKA. Their functions included glucose metabolism (*HXK1, YIG1*), lipid metabolism (*CRC1, INO1, YAT1, YAT2*), nitrogen metabolism (*ATO3, PUT1, PUT4*), phosphate metabolism (*PHO89*), and tolerance to high temperature (*HSC82, HSP10, HSP42, HSP60, SPG4*). Interestingly, many genes that were repressed by glucose in a PKA-negative background are known to be controlled by the transcription factor Cat8 (*ACS1, ADY2, ATO3, FBP1, ICL1, JEN1, MDH2, MLS1, ODC1, PCK1, PUT4, SFC1, YAT2*) [22] or to be direct targets of Ume6 (*ACS1, ADY2, INO1, SIP4*) [23] or Hap1 (*CYB2, HXM1, MLS1, PUT1, PUT4*) [24].

Of the genes that are no longer or hardly repressed by glucose, in the absence of PKA, many are related with respiration (*COQ5, COX8, CYC3, CYT1, SDH1/2/3/4*), ATP synthesis (*ATP11, ATP14, ATP18, STF2*), or stress responses (*CTA1, DDR2, GRX2, HSP12, HYR1, PRX1, PST2, TPS1, TSA1, TSL1, TRX1*). Others are involved in carbon metabolism (*ALD4, ARA1, GPD1, HXT5/6/7, UGP1*) or nitrogen metabolism, often in relation with proteolysis, (*ALD3, AGX1, LAP4, PAI2, PBI2, PEP4, PRC1, RBD2*).

### Effects of PKA on repression by glucose during exponential growth

Most of the genes repressed by glucose in the short-term experiments, particularly genes required for the utilization of alternative carbon sources, are known to be fully repressed during exponential growth on glucose [25]. We wondered whether genes involved in other cellular processes are subject to both short-term and long-term glucose repression. To address this question we selected some genes whose repression is strongly dependent on PKA. As shown in Table 2, we found that for *TPS1 *and *TPS2*, that encode the catalytic subunits of the trehalose-6-phosphate synthase/phosphatase complex, repression was transient and the same was observed for *GRX2 *and *PEP4*. In contrast, the *HXT6/7 *genes were still strongly repressed during growth, in a PKA-dependent manner.

**Table 2:** Short-term vs. long-term effects of lack of PKA on repression of different genes by glucose

Strain	Growth conditions	Gene				
		
		*TPS1*	*TPS2*	*HXT6/7*	*GRX2*	*PEP4*
		
		Repression factor				
TPK1 TPK2 TPK3	30 min with glucose	7.7	7.1	30	9.5	15
	
	growth in medium with glucose	1.3	1.6	68	2.9	1.8

*tpk1 tpk2 tpk3* *msn2 msn4*	30 min with glucose	1.3	1.1	0.2	0.7	0.4
	
	growth in medium with glucose	1.5	1.1	0.2	0.9	1

*tpk1 tpk2 tpk3* *yak1*	30 min with glucose	1.1	1.3	1.4	1.4	1.2
	
	growth in medium with glucose	0.3	0.4	0.3	0.5	0.6

Yeasts were grown on ethanol as described in Methods and samples were taken before the addition of glucose to the medium and 30 min after. The yeasts were also grown on a medium containing 2% ethanol and 4% glucose and collected when at least 50% of the glucose was still present in the medium. Biological duplicates were performed and mRNA levels were measured by RT-PCR as described. Expression data from biological replicates generally differed from the average by less than 25%. The repression factor is the quotient of the values measured in samples grown in the absence of glucose and in the presence of glucose.

### Impact of PKA on transcript profiles in gluconeogenic cultures

During growth under gluconeogenic conditions, most yeast genes were expressed at similar levels in the *TPK1 TPK2 TPK3 *reference strain and in the two strains that lack a functional PKA. There was, however, a set of genes expressed at reduced levels in both mutant strains, and this set showed an overrepresentation of genes involved in stress responses, including thermal or oxidative stress (*GPH1, GRE1, HSP26, HSP30, PMA2, SPG1, SPG4, YRO2, GAD1*), osmotic shock (*SIP18, STL1*), and DNA damage (*DDR48, YNL194c*). Reduced expression of these genes was observed in both the *msn2 msn4 *and the *yak1 *genetic backgrounds, thus supporting the interpretation that PKA is required for their full expression under gluconeogenic conditions. We checked that most of these genes (except for *DDR48, HSP30, SPG4, YNL194c*) were also poorly expressed in a *tpk^w ^bcy1 *strain of *S. cerevisiae *grown under gluconeogenic conditions [5], an observation consistent with a dependence on PKA.

Although most genes showed a similar transcript level in the *tpk1 tpk2 tpk3 msn2 msn4 *and *tpk1 tpk2 tpk3 yak1 *strains, some notable exceptions were observed (Table 3). For the majority of genes expressed at lower levels in the *tpk1 tpk2 tpk3 msn2 msn4 *strain during growth on ethanol this is easily explained, as their expression has previously been reported to be activated by Msn2/4 [3]. The three remaining genes of this category (*NCA3, STF2, YKL187c*) are involved in some stress responses and have a mitochondrial function, but have no previously established relation with Msn2/4. Nevertheless, their behaviour is consistent with an as yet unidentified, and perhaps indirect, regulation by Msn2,4. For genes expressed at higher levels in the *tpk1 tpk2 tpk3 msn2 msn4 *strain or at higher or lower levels in the *tpk1 tpk2 tpk3 yak1 *strain no immediate explanation is apparent.

**Table 3:** Genes with different expression in strains *tpk1 tpk2 tpk3 msn2 msn4 *and *tpk1 tpk2 tpk3 yak1 *during growth on a gluconeogenic carbon source

Gene	WT	*tpk1 tpk2 tpk3 msn2 msn4*	*tpk1 tpk2 tpk3 yak1*
HXK1*	459	77	228

HSP104*	327	121	229

MSC1*	629	58	296

SOL4*	148	28	107

*YKL187c*	321	28	124

GLC3*	390	96	425

GLK1*	852	465	889

GPX1*	271	41	196

GSY2*	184	45	245

HSP12*	3790	807	2436

HPF1*	650	137	611

*NCA3*	744	134	683

SOD1*	1964	841	1558

*STF2*	838	183	1004

TPS1*	569	245	612

TSL1*	274	45	245

*ATF2*	31	33	173

*PRM5*	116	339	164

*SSA2*	997	1519	611

*DAL3*	198	403	58

*FSH1*	220	271	68

Yeast strains *TPK1 TPK2 TPK3, tpk1 tpk2 tpk3 msn2 msn4*, and *tpk1 tpk2 tpk3 yak1*, were grown on ethanol and samples were taken before the addition of glucose to the medium, as described in Methods. mRNA levels were measured using Affymetrix microarrays. Biological duplicates were performed. Expression data from biological replicates generally differed from the average by less than 20%. Expression is shown in arbitrary units. (* Genes known to be activated by Msn2/4).

## Discussion

To bypass the lack of viability of *S. cerevisiae *strains lacking PKA, we have used strains lacking PKA and bearing suppressor mutations. Other, indirect, approaches have previously been used to study if PKA, or activation of PKA by cAMP, is required for the response of yeast to glucose. Slattery et al [26] used a *cyr1*Δ strain, lacking adenylate kinase and therefore requiring cAMP for growth; the strain was grown in YPD + cAMP, starved for 24 h in spent YPD, resuspended in fresh YPD in the presence or absence of cAMP, and the changes produced in the transcriptome in these two conditions were compared. A different method was used by Zaman et al [27] to study the effects of blocking glucose signalling through PKA. In that work a *tpk1^as ^tpk2^as ^tpk3^as ^*strain was built, with modified Tpks that had become sensitive to the inhibitor 1NM-PP1. Glucose was then added, together with the inhibitor, to a wild-type strain and to the mutant strain, both grown under gluconeogenic conditions, and the transcriptional responses of the two strains were compared. Our approach presents the advantage over the other two methods of allowing long-term experiments with growing cells (such as those shown in tables 1 and 2). It may also be noted that if phosphorylation of some protein by PKA, taking place in the absence of glucose, played a role in the transcriptional response to glucose, this would not be detected in the experiments performed by Zaman et al [27] where PKA activity was only blocked when glucose and the specific inhibitor were added to the cells. While avoiding the disadvantages associated with the use of inhibitors, the use of suppressor mutants can also present its own problems. In particular, the suppressor mutations may interfere with pathways related to the normal cellular responses to glucose or activate pathways that are not implicated in the response to glucose in wild-type backgrounds. In the present study, we have sought to minimize the risk of such complications by using strains with two different suppressor mutations.

Our results in the short-term experiments are generally consistent with the data presented by Zaman et al [27]. The main differences were found in some groups of genes induced by glucose. In our experiments the transcript levels of genes involved in glycolysis (*PFK1, ENO1, ENO2, CDC19, ADH1*) showed a small increase (40-80%) both in the reference strain and in the strains lacking PKA. In contrast, Zaman et al [27] found that these genes were repressed by glucose in the reference strain (2 to 6-fold). In their experiments blocking PKA eliminated this repression, allowing a slight induction (20-80%) in some cases. Induction by glucose of genes related with ribosome biogenesis in the reference strain was similar in the two sets of experiments; however, in our conditions most of these genes were induced in the absence of PKA, while in the work of Zaman et al. [27] inhibition of PKA impaired their induction. Since *S. cerevisiae *strains with the W303 genetic background were used in both studies, it seems likely that the disparities observed are related with the different media used for growth. Zaman et al. [27] used glycerol and ammonium as carbon and nitrogen source while our cultures contained ethanol and glutamate.

As shown in Tables 1 and 2, for a number of genes the transcriptional response to glucose is transient. This may be explained by the fact that the large increase in intracellular cAMP, sometimes referred as the cAMP "spike", observed upon exposure of the yeast cells to glucose is itself transient [28]. It could be expected that for genes that respond to glucose independently of PKA the response would be long-lived and, in fact, induction of *PDC1 *is maintained or even increased during exponential growth (Table 1). For *ALD5, TMT1, YMC2 *and *ATR1 *two signalling pathways appear to be operative, one of them independent of PKA and acting both shortly after glucose addition and during growth, the other one depending on PKA and with transient effects. The behaviour of *PDC5 *could be explained by the operation of a PKA-independent system whose effects are transient. We have also observed that for some genes, that show no response to the sugar in the absence of PKA, repression by glucose in the wild-type strain is transient (Table 2).

Our observations on the involvement of PKA in induction and repression by glucose, together with the results of Wang et al [5], allow the classification of genes responding to glucose in four categories (Table 4).

**Table 4:** Involvement of PKA in the transcriptional regulation of different classes of genes controlled by glucose

Class	Transcriptional effect	Glucose added to strain		No glucose added	Representative genes
				
		PKA^+^	PKA^-^	PKA activated	
1	Induction	+	+	-	*PFK1, ENO2*
	Repression	+	+	-	*CRC1, PCK1, MLS1*
2	Induction	+	-	+	*ARG3, THR4*
	Repression	+	-	+	*GRX2, PEP4*
3	Induction	+	+	+	*HXT3, RPL7A, HOR2*
	Repression	+	+	+	*ACS1, ADY2, CIT3*
4	Induction	+	-	-	*SDT1, STR3*
	Repression	+	-	-	*HXT6, MDH1*

The presence or absence of transcriptional response after addition of glucose to yeast strains with or without PKA activity is indicated by + or -. The column labelled "No glucose added" shows the transcriptional response when PKA is activated through an increase in cAMP that does not involve the presence of glucose [5].

### Genes in class 1 are controlled exclusively by signalling pathway(s) independent of PKA

These genes can be induced (or repressed) in the absence of PKA and are not induced (or repressed) upon activation of PKA in the absence of glucose. In this category, induced genes are mostly related with glucose metabolism, while repressed genes are involved in utilization of alternative carbon sources.

#### Genes in class 2 are controlled only by signalling pathway(s) dependent on PKA

These genes can be induced/repressed by activation of PKA and do not respond to glucose in the absence of PKA. Many of the induced genes in this category encode proteins involved in amino acid biosynthesis, while the repressed genes form a heterogeneous group.

#### Genes in class 3 are controlled by redundant PKA-dependent and PKA-independent signalling pathways

These genes can be fully induced/repressed by activation of PKA, but also by glucose in the absence of PKA. Among the induced genes in this category, those encoding glucose transporters and ribosomal proteins are prevalent, while many of the repressed genes are involved in the utilization of non-fermentable carbon sources.

#### Genes in class 4 are controlled by cooperative signalling pathways, of which at least one is PKA-dependent

These genes encode proteins of different types. Some of them do not respond to the activation of PKA nor to the addition of glucose in the absence of PKA. For others, a partial induction/repression may be observed in one of these conditions or in both.

What could be the advantages to the cell of employing these different modes of regulation? While control through a single signal may be adequate in many cases, regulation of genes by redundant signalling pathways could confer a greater robustness to their response to glucose. On the other hand, regulation by several cooperative pathways that, individually, cannot give a full transcriptional response, could provide a greater flexibility, as discussed by Chen and Thorner [29] for the case of yeast responses to nutrient limitation.

The next important goal will be to establish the PKA-dependent and PKA-independent pathways operating in each case, since information on this topic is still very incomplete. A notable exception is the PKA-independent pathway controlling the glucose-induced *HXT *genes that encode glucose transporters. The plasma membrane proteins Snf3/Rgt2 act as sensors for external glucose and, when bound to the sugar, initiate a chain of reactions that results in the dissociation of the repressor Rgt1 from the promoters of the corresponding *HXT *genes [30,31]. For other types of genes induced by glucose the situation is less clear. The protein kinase Snf1 may play a role in the case of genes encoding enzymes involved in amino acid biosynthesis, since Snf1 represses the translation of the transcriptional activator Gcn4 [32]. As glucose inactivates Snf1 [33], this would result in the induction of the genes activated by Gcn4. Another important group of glucose-induced genes encodes proteins involved in the biogenesis of ribosomes and is subject to a very complex combinatorial control. Binding, through Rap1, of the transcription factor Fhl1 together with the coactivator Ifh1 to the corresponding promoters activates the transcription of these genes, while other factors, Cfr1, Dot6 and Tod6, Stb3, contribute to repress their transcription. Glucose modifies the intracellular localization of these factors: it promotes a cytoplasmic location for Cfr1 [34], Dot6 and Tod6 [35], and Stb3 [36] and the nuclear localization of Fhl1 and Ifh1 [37]. While PKA can phosphorylate Dot6 and Tod6 and this is likely to direct them to the cytoplasm, export from the nucleus of Stb3 depends on Tor activity but not on PKA. Glucose also promotes the phosphorylation by PKA of the global transcriptional regulator Sfp1, responsible for the nuclear relocalization of Fhl1 and Ifh1. However, phosphorylation by PKA of Sfp1, in the absence of glucose, is not sufficient to direct Sfp1 to the nucleus [37]. This indicates that an alternative signalling pathway is involved in the process; it may be Tor-dependent, since there is already evidence for activation by glucose of the Tor signalling pathway [36,38,39]. However, the molecular mechanism responsible for this effect of glucose on Tor has not been yet established. With respect to genes encoding glycolytic enzymes, they depend on the transcription factors Gcr1 and Gcr2 [40,41] but there is no information on the regulation of these factors by glucose.

Most of the genes repressed by glucose depend for their transcription on the activity of the protein kinase Snf1 and/or on the transcription factors Hap1, Hap4, Cat8 and Adr1 [27]. There is no evidence that Hap1 itself is regulated by glucose; repression by glucose of genes activated by Hap1 depends on its control of other transcription factors such as Hap4 or Adr1 [42]. Transcription of *HAP4 *and *ADR1 *is repressed by glucose and we have found that the degree of repression decreases in the absence of PKA, but the intimate mechanism of the repression of transcription is unknown. Snf1 is not required for the induction of *ADR1 *[43] or of *HAP4 *[27], that takes place in the absence of glucose, but a Snf1-dependent pathway is responsible for the activation of the transcription factor Adr1 in these conditions [44]. Snf1 is also necessary both for the transcription of *CAT8 *and for the transcriptional activation of Cat8 [45]. We have observed that glucose represses *SNF1 *transcription about 2-fold, in addition to its well-known role in decreasing Snf1 activity [33]. Although the mechanism for this decrease is not established, it has been proposed that the β subunit of the Snf1 complex is important to maintain Snf1 in an inactive state [46]. Specifically, the glycogen-binding domain of the β subunit Gal83 would mediate the recruitment of the Reg1-Glc7 complex, responsible for the dephosphorylation and subsequent inactivation of Snf1.

It should be stressed that, even in cases where PKA has been shown to play an important role in the control of gene transcription by glucose, the target for PKA has not been generally identified. A possible approach for clarifying the role of PKA could be to check whether selected proteins identified as potential PKA substrates participate in the glucose response. Surprisingly, among the many proteins interacting with Tpk1, Tpk2 or Tpk3 and characterized as possible substrates [47,48] few good candidates appeared. The proteins Msn2/Msn4 are an interesting exception. For the genes under control of Msn2/Msn4, PKA operates directly through the phosphorylation of the transcription factors, allowing their export to the cytoplasm, and thus precluding transcription. As shown in table 5, expression of the relevant genes is already low in the absence of glucose in the strain lacking Msn2/Msn4 and the effect of glucose is modest. In the *tpk1 tpk2 tpk3 yak1 *strain expression is similar to that of the wild-type strain under gluconeogenic conditions but it is much less sensitive to the addition of glucose, as the transcriptional activators remain in the nucleus. New strategies would have to be devised to identify additional protein substrates of PKA that may play a role in the response to glucose.

**Table 5:** Genes activated by Msn2/Msn4 and depending on PKA for glucose repression

Gene	Relevant genotype					
	
	*TPK1 TPK2 TPK3*		*tpk1 tpk2 tpk3 msn2 msn4*		*tpk1 tpk2 tpk3 yak1*	
	
	Et	+ Glu	Et	+ Glu	Et	+ Glu
*GPX1*	100	6	15	8	73	45

*GSY2*	100	13	23	15	80	62

*HPF1*	100	41	21	42	94	108

*MSC1*	100	4	9	6	45	39

*TSL1*	100	8	16	9	90	92

A wild-type strain and two strains lacking PKA were grown on ethanol, samples taken before (Et) and 30 min after the addition of glucose (+Glu), and mRNA levels measured (see Methods for details). Expression is given as percent of the value in the wild-type strain growing on ethanol.

## Conclusions

This study shows that, although PKA is frequently involved in the control of transcription by glucose, there are many instances in which PKA activity is dispensable. We have also observed that for genes regulated exclusively through PKA the effect of glucose on transcription may be transient. It may be concluded that the role of cAMP/PKA in transcription, which was first identified a couple of decades ago, still represents many challenges and that one of the goals of systems biology, the construction of predictive models of signal transduction pathways, requires additional detailed studies on the mechanism(s) by which PKA is involved in the transcriptional regulation of a substantial part of the yeast genome and of the context dependency of these mechanisms.

## Methods

### Yeast strains and growth conditions

The yeast strains used are listed in Table 6. All of them are derived from strains W303-1A and W303-1B [49]. The prototrophic strains CJM 567, CJM 573, CJM 569 and CJM 571 were constructed by substituting in the original strains, W303-1A, W *yak1 *[14], W303 *tpk123 msn2 msn4 *[50] and W303 *tpk123 yak1 *[51], through successive transformations, the mutated genes (*ade2, his3, leu2, trp1, ura3*) by their wild-type alleles, as needed, using selective media. The mutant strains, CJM 573, CJM 569 and CJM 571 were checked by diagnostic PCR.

**Table 6:** *S. cerevisiae *strains used in this work

Strain	Genotype	Reference
W303-1A W303-1B	*MATa ade2-1 his3-11,15 leu2-3, 112 trp1-1 ura3-1 can1-100 TPK1 TPK2 TPK3* *MATα ade2-1 his3-11,15 leu2-3, 112 trp1-1 ura3-1 can1-100 TPK1 TPK2 TPK3*	[49] [49]
W *yak1*	*MATα ade2-1 his3-11,15 leu2-3, 112 trp1-1 ura3-1 can1-100 yak1::KanMX TPK1 TPK2 TPK3*	[14]
W *tpk1 tpk2 tpk3 msn2 msn4*	*MATa ade2-1 his3-11,15 leu2-3, 112 trp 1-1 ura 3-1 can1-100 tpk1::URA3 tpk2::HIS3 tpk3::TRP1 msn2::HIS3 msn4::TRP1*	[50]
W *tpk1 tpk2 tpk3* *yak1*	*MATα ade2-1 his3-11,15 leu2-3, 112 trp1-1 ura3-1 can1-100 tpk1::URA3 tpk2::HIS3 tpk3::TRP1 yak1::LEU2*	[51]
CJM567	*MATa can1-100 TPK1 TPK2 TPK3*	This work
CJM573	*MATα can1-100 yak1::KanMX TPK1 TPK2 TPK3*	This work
CJM569	*MATa his3-11,15 ura3-1, 112 trp1-1 can1-100 tpk1::URA3 tpk2::HIS3 tpk3::TRP1 msn2::HIS3 msn4::TRP1*	This work
CJM571	*MATα his3-11,15 leu2-3, 112 ura3-1 trp1-1 can1-100 tpk1::URA3 tpk2::HIS3 tpk3::TRP1 yak1::LEU2*	This work

Yeasts were grown at 30°C in minimal media (YNB with 0.1% sodium glutamate as nitrogen source) with 2% ethanol or 4% glucose + 2% ethanol as carbon sources and collected at the early exponential phase of growth. Samples were also taken after adding glucose (end concentration 4%) to cultures in 2% ethanol and incubating them at 30°C for 30 min.

### Sampling and RNA isolation

Yeast cells were collected by rapid filtration [52] and stored at -70°C until use. The RNA to be used for the microarrays was extracted using the hot-phenol method [53]. For the RT-qPCR measurements RNA was extracted with glass beads and the TRIzol LS reagent (Invitrogen, Carlsbad, CA, USA) as described [52] and quantified using the Thermo Scientific NanoDrop Spectrophotometer.

### Probe preparation and hybridization to arrays

cDNA synthesis, cRNA synthesis and labelling, as well as array hybridization were performed as described [54].

### Data acquisition and primary analysis

Acquisition and quantification of the Affymetrix yeast S98 array images as well as primary data analysis were performed using the Affymetrix GeneChip Operating Software (GCOS) 2.1. All arrays were globally scaled to a target value of 150 using the average signal from all gene features. The Significance Analysis of Microarrays (SAM version 3.0) add-in to Microsoft Excel was used for comparisons of duplicate array experiments [55] SAM assesses the difference between two mean values when taking into account the standard errors of those means. The significance of that difference is estimated by comparing it against the probability of its occurrence by chance alone. The model of chance occurrence is generated by permutation of the input data, rather than a predetermined model (*e.g*. a normal distribution), as is used by the *t*-test.

The transcript data can be downloaded from Genome Expression Omnibus (http://www.ncbi.nlm.nih.gov/geo) under the series accession number GSE27541.

### RT-qPCR measurements

The RNA samples were treated with DNase I (Ambion) and the quality of the purified RNA checked using the Agilent 2100 Bioanalyzer. The oligonucleotides used for RT-qPCR are shown in table 7. The measurements were performed in triplicate using SYBR green I. Normalization was carried out with the 18S rRNA.

**Table 7:** Probes used for the RT-qPCR measurements

Gene	Direct oligonucleotide	Reverse oligonucleotide	Product(bp)
*PDC1*	TAGAACTCCAGCTAACGCTGCTGT (1053-1076)	TGGGAAAGTGGTTTGGTTGATACC (1180-1203)	151
*PDC5*	GCCAAGTCAACTGTAACACCGTCT (44-68)	CAGCATAGGCAGCGTTCAATTCGT (149-173)	130
*HXT6*	GGGCTGTTTGGTCTTCATGTTCTTC (1497-1521)	CATTTCTTCAGCGTCGTAGTTGGC (1642-1665)	169
*ALD5*	AGCCAACAGTGTTTGCTGATGTCAA (1205-1229)	GCGGCTAACCCATATTGAGAATCAT (1322-1346)	142
*TPS1*	TCTCGTCCACCCGTGATGGTATGAA (1301-1325)	ATGGCATCAGAAAGATCATCGGTGT (1160-1164)	166
*TPS2*	GGGCAACTACGGATTCTATCCTGTC (2430-2454)	TCTGGAATCCAGGTCGACCGTACCA(2568-2592)	163
*ATR1*	TTATTAGTAGAGCCTTCCAAGGGC (494-517)	CAACCTAAAGTTCGTCCAATAGGG (601-647)	156
*GRX2*	CTACTCCAAAAATGGTATCCCAGG (92-115)	GGAACGTTCAATTCTTGGAAGAGG (210-233)	144
*PEP4*	GATAAGGTGGTCCCTCCATTTTAC (622-645)	AACAGGTAACCAAGTGATATCGCC (769-792)	173
*TMT1*	TGATTGAAGTTCCTTACGGGAAGC (494-517)	CTTTGTCTCTCACATCTTCTGCAC (629-652)	161
*YMC2*	GTTGACAGTATACCCATTGGACGTT (762-786)	CCAAACCCTTTGAAAAAGGCTCTGA (881-905)	146

The oligonucleotides were checked for specificity using the BLAST feature of SGD.

## List of abbreviations

PCR: polymerase chain reaction; PKA: protein kinase A; RT-qPCR: quantitative real-time polymerase chain reaction; YNB: Yeast Nitrogen Base.

## Authors' contributions

DL performed the cultures, collected the yeast samples and extracted RNA. MA performed the microarray experiments. JMD analysed the microarray data. JTP co-designed the experiments and contributed to the writing. JMG conceived the study, designed experiments, constructed yeast strains, analysed data and wrote the paper. All authors discussed the results and read and approved the final manuscript.
